# Evaluation of Axial Compressive and Tensile Properties of PE/PVA Hybrid Fiber Reinforced Strain-Hardening Geopolymer Composites

**DOI:** 10.3390/ma17174356

**Published:** 2024-09-03

**Authors:** Jingen Guo, Ji Shi, Liuhuo Wang, Chengyong Huang, Xiongwu Tao, Chaosen Li, Zhanbiao Chen

**Affiliations:** 1 Huizhou Power Supply Bureau, Guangdong Power Grid Co., Ltd., Huizhou 516001, China; guo19830526@163.com (J.G.); 13829984272@139.com (J.S.); firehuo_0403@126.com (L.W.); 13502201000@139.com (C.H.); 13500175094@139.com (X.T.); 2 China Southern Power Grid Co., Ltd., Guangzhou 510663, China; 3 Guangdong Power Grid Co., Ltd., Grid Planning Research Center, Guangzhou 510050, China; 4 School of Civil and Transportation Engineering, Guangdong University of Technology, Guangzhou 510006, China; yyyee09007@126.com

**Keywords:** SHGC, non-oil-coated PVA fiber, hybrid, compressive, tensile, cost analysis

## Abstract

The strain-hardening geopolymer composite (SHGC) is a new type of fiber concrete with excellent ductility and environmental friendliness. However, the high cost of fibers greatly limits its widespread application. This paper proposes the use of untreated low-cost polyvinyl alcohol (PVA) fibers and polyethylene (PE) fibers to develop a low-cost, high-performance SHGC. Axial compression and axial tension tests were conducted on the SHGC with different PE fiber volume fractions (1%, 1.5%, and 2%) and different PVA fiber replacement ratios (0%, 25%, 50%, 75%, and 100%) to investigate the hybrid effects of fibers with different surface properties and to reveal the mechanism of fiber hybridization on the mechanical behavior of SHGCs. The results show that increasing the PE fiber volume fraction improves the compressive and tensile ductility of the SHGC while increasing the PVA fiber replacement rate impacts the strength indicators positively due to the good interface effect formed between its hydrophilic surface and the matrix. When the PVA fiber replacement ratio is 100%, the compressive strength (93.4 MPa) of the SHGC is the highest, with a 21.1% increase compared to the control group. However, the tensile strength shows a trend of first increasing and then decreasing with the increase in the PVA fiber replacement ratio, reaching the highest at a 25% replacement ratio, with a 12.5% increase compared to the control group. Furthermore, a comprehensive analysis of the economic and environmental performance of the SHGC indicates that a 25% PVA fiber replacement ratio results in the best overall economic benefits and relatively low actual costs, although the effect of fiber hybridization on carbon emission indicators is not significant. This paper provides new ideas and a theoretical basis for designing low-cost SHGCs.

## 1. Introduction

Concrete is widely used due to its advantages, such as its ease of pouring and convenient sourcing. However, the cement used as a binding material emits a significant amount of carbon dioxide during its production process [1,2,3], causing serious environmental harm. Therefore, finding alternative materials is particularly urgent. Geopolymer is a novel binding material obtained by the alkaline activation of industrial solid wastes rich in silicon and aluminum (such as fly ash (FA), ground granulated blast furnace slag (GGBS), and red mud (RM) [4,5,6]). This material features high compressive strength, good impermeability, and excellent chemical resistance [7,8,9]. Moreover, its production process reduces carbon emissions by 80% compared to cement [10], making it a promising alternative. However, concrete produced with geopolymers remains brittle and has limitations in practical applications [11]. Current research indicates that adding materials such as rubber aggregates [12], nano-additives [13,14], and fibers [15,16] appears to be an effective method for mitigating the brittleness of concrete.

In recent years, scholars have developed a new type of fiber-reinforced concrete, called strain-hardening cementitious composite (SHCC), based on micromechanics principles [17,18,19]. Under tensile loading, this new concrete can achieve ultimate tensile strains of over 3%, with crack widths limited to less than 100 μm [20,21,22]. The application of this new concrete effectively addresses the brittleness issue commonly found in ordinary concrete. Leveraging the micro-design criteria of the SHCC, combining low-carbon geopolymers and high-performance fibers appears to be a promising approach to mitigate brittleness in geopolymers and reduce carbon emissions in the construction industry. Shaikh [23] first proposed the use of fly ash-based geopolymers as a complete substitute for cement to develop high-ductility strain-hardening geopolymer composites (SHGCs) in 2013, which showed even better deformability than SHCCs. Subsequently, numerous scholars have researched and optimized the performance of SHGCs [6,24,25,26,27]. Ohno and Li [28] utilized oil-coated polyvinyl alcohol (PVA) fibers and a fly ash-based geopolymer matrix to prepare a SHGC with a compressive strength reaching 27.6 MPa and tensile strain capacity of 4.3%. Lao et al. [29] employed ultra-high molecular weight polyethylene (UHMWPE) fibers, a slag-fly ash geopolymer matrix, and seawater sea sand to develop a seawater sea sand SHGC, achieving a compressive strength and ultimate tensile strain of over 140 MPa and 8%, respectively.

The trade-off for improving the mechanical properties of SHGCs is their high cost and high carbon footprint. The currently developed SHGC typically requires high-performance synthetic fibers such as oil-coated PVA fibers and PE fibers [20,30,31]. These high-performance fibers are expensive and have a significant carbon footprint [32], which limits the widespread adoption and application of SHGC materials. For example, oil-coating treatment on PVA fibers can reduce the chemical bonding between PVA fibers and the matrix, thereby enhancing the tensile deformability of SHCCs [33]. However, this process significantly increases the price of PVA fibers, making their proportion in the total material cost of a SHGC as high as 70% [32]. Therefore, reducing the fiber costs is an effective way to improve the economic feasibility of the SHCC/SHGC. Zhang et al. [34] used non-oil-coated PVA fibers to prepare a SHCC in an attempt to lower the material costs. Tensile tests comparing oil-coated PVA and non-oil-coated PVA on SHCCs showed that using non-oil-coated PVA reduced the tensile strength and deformability of the SHCC but still exhibited some strain-hardening capability. Furthermore, considering the hybrid use of low-cost fibers (for example, regenerated fibers such as polyethylene terephthalate (PET), polyamide (PA), and polypropylene (PP) [35]) with high-performance fibers can effectively reduce SHCC/SHGC costs [36,37,38,39,40]. Yu et al. [38] studied a typical SHCC (M45 in Li [41]) and found that replacing the PVA fibers with different proportions of recycled PET fibers negatively affected the tensile properties of the SHCC to varying degrees but improved its economic and environmental sustainability. Lin et al. [31] investigated the tensile mechanical behavior of a SHGC by replacing the PE fibers with different proportions of PP fibers. They observed a decrease in tensile strength with the use of PP fibers, but the ultimate tensile strain showed optimal performance at a 50% replacement ratio (9.71%), effectively enhancing the economic benefits of SHGCs.

However, current research on preparing SHGCs with hybrid fibers mostly leans towards using fibers with similar interface properties, which still has certain limitations. There is considerable room for improvement in the design of low-cost SHGCs. This study designs a SHGC using untreated low-cost PVA fibers mixed with high-performance PE fibers. Axial compressive and tensile tests were conducted on a SHGC with varying PE fiber volume fractions (1.0%, 1.5%, and 2.0%) and PVA fiber replacement ratios (0%, 25%, 50%, 75%, and 100%) to investigate the hybrid effects and reinforcement mechanisms of fibers with different surface characteristics (hydrophilic PVA fibers and hydrophobic PE fibers) on the mechanical behavior of a SHGC. Additionally, a comprehensive analysis of the economic and environmental benefits of the SHGC under different fiber parameters was performed. This study aims to provide reliable experimental data and theoretical foundations for designing cost-effective, high-performance SHGCs.

## 2. Experimental Program

### 2.1. Materials

**Figure 1:**
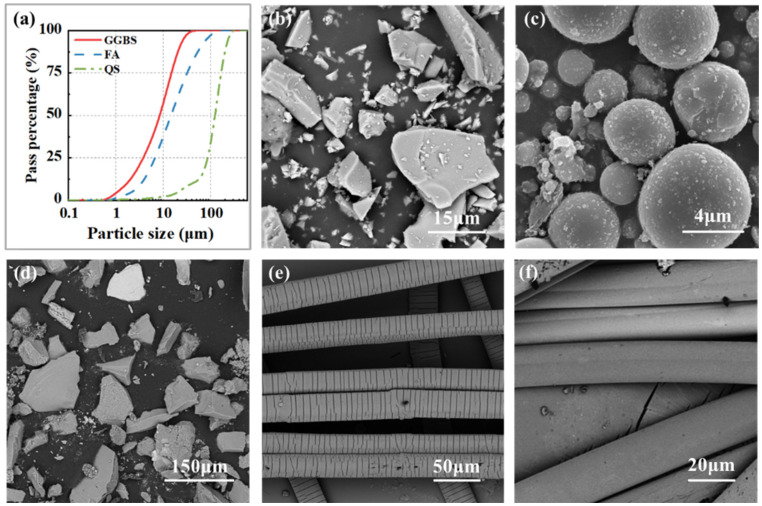
(**a**) Particle size distribution curves of GGBS, FA, and quartz sand; (**b**–**f**) microscopic images of raw materials. (**b**) GGBS; (**c**) FA; (**d**) quartz sand; (**e**) PE fibers; (**f**) PVA fibers.

**Figure 2:**
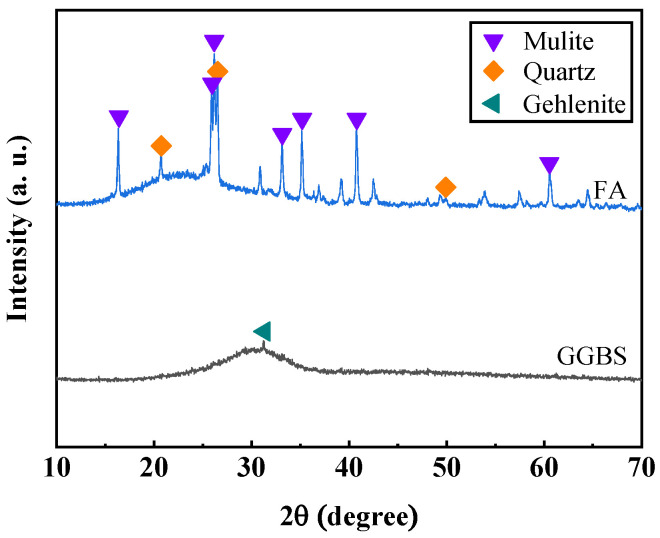
XRD patterns of GGBS and FA.

SHGC’s basic mix proportion referenced prior research by Lin et al. [31] and is primarily composed of precursors, quartz sand, alkaline activators, retarders, and synthetic fibers. The precursors consist of ground granulated blast furnace slag (GGBS) and fly ash (FA), classified according to the GB/T 18046 [42] and ASTM C618 [43] standards, with the GGBS categorized as an S105 grade and FA as Class F. Figure 1a shows the particle size distribution of slag, fly ash, and quartz sand, which exhibits a characteristic of “overall wide distribution with local narrow distribution”. This ensures good fluidity of the fresh matrix and good density of the hardened SHGC. Table 1 shows the chemical composition of the GGBS and FA, with the GGBS providing a significant amount of calcium, thereby ensuring the strength of the SHGC. Alkali-activator comprises sodium hydroxide solution (concentration: 10 mol/L) and sodium silicate solution (modulus: 2.25, the mass ratio of SiO_2_, Na_2_O, and H_2_O is 29.99:13.75:56.26), facilitating geopolymerization. BaCl_2_ serves as a retarder, delaying the setting time of fresh SHGC paste to meet the workability requirements. The synthetic fibers used in this study include PE fibers and non-oil-coated PVA fibers, characterized by their physical parameters listed in Table 2. These data are sourced from the raw material manufacturer. Figure 1b–f shows the microscopic images of the raw materials, such as the precursors, quartz sand, and synthetic fibers. The equipment used for the scanning electron microscope (SEM) analysis was the Phenom Pharos G2, produced by Phenom-World B.V. (Eindhoven, the Netherlands). FA particles are spherical; thus, their incorporation can produce a “ball bearing effect”, which positively contributes to the flowability of the SHGC. Figure 2 shows the XRD patterns of the GGBS and FA. The patterns indicate that the main crystalline phase of the GGBS is gehlenite, while the primary crystalline phases of FA are mulite and quartz. Additionally, both the GGBS and FA exhibit broad, amorphous peaks in the XRD patterns, which suggests that they possess good reactivity.

### 2.2. Mix Proportions and Specimens

**Figure 3:**
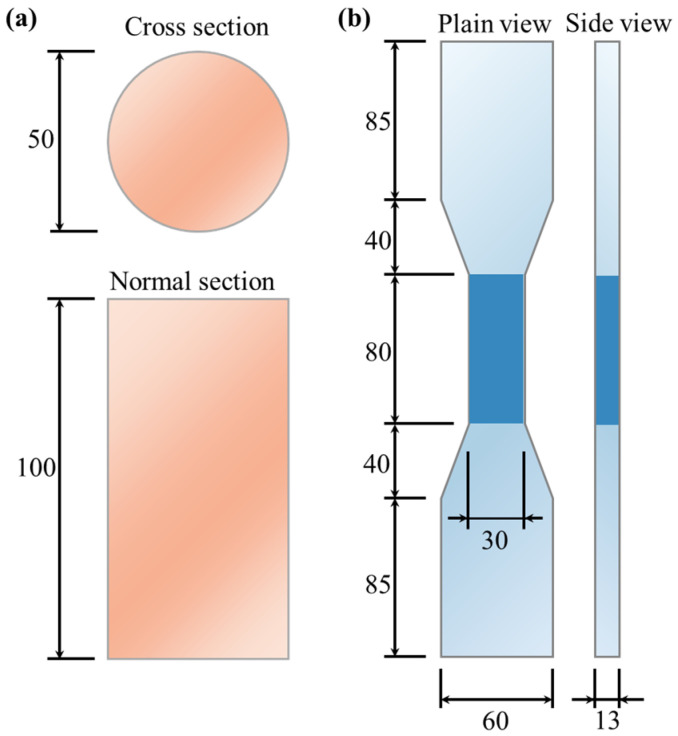
Experimental specimen dimensions (unit: millimeter, mm). (**a**) Cylinder specimen; (**b**) dumbbell-shaped specimen.

This study employed cylindrical and dumbbell-shaped specimens for the axial compressive and tensile tests, respectively, with the specific dimensions shown in Figure 3. The study variables included PE fiber volume fractions (1.0%, 1.5%, and 2.0%) and PVA fiber replacement ratios (0%, 25%, 50%, 75%, and 100%). PVA fibers were used to replace PE fibers by volume, with a fixed fiber volume fraction of 2%. The specific mix proportions are detailed in Table 3.

**Figure 4:**
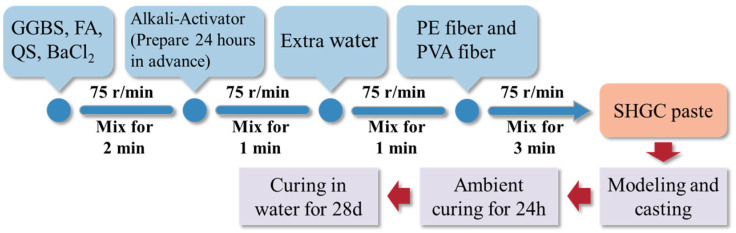
Process of SHGC specimen preparation.

The specimen preparation process is as follows: initially, GGBS, FA, quartz sand, and BaCl_2_ are added to a planetary mixer and stirred at a speed of 75 rpm for 2 min. Then, alkaline activators are added and stirred at 75 rpm for 1 min (notably, the alkaline activator, composed of sodium hydroxide solution and sodium silicate solution, needs to be mixed and cooled for 24 h before use). After thorough mixing, additional water is added and stirred at 75 rpm for 1 min. Finally, the PE and PVA fibers are sequentially added and stirred for 3 min to obtain the SHGC paste. The fresh paste is poured into prepared molds, covered with plastic film, and cured at room temperature for 24 h before being transferred to a water tank for further curing for 28 days. Figure 4 illustrates the specimen preparation process.

### 2.3. Test Methods

According to ASTM C1437 [44], the flowability of the fresh SHGC pastes was tested. Using a steel ruler, the diameters in two perpendicular directions of the spread paste were measured to determine its flowability.

**Figure 5:**
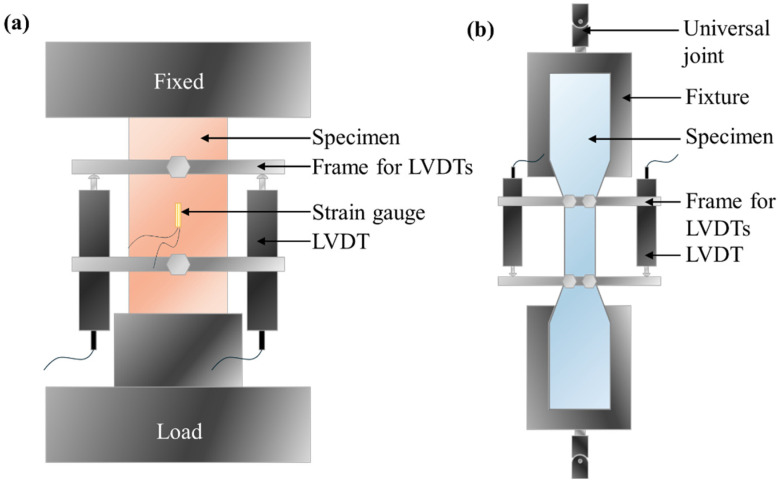
Experimental setup: (**a**) axial compressive test; (**b**) axial tensile test.

For the axial compressive test, following ASTM C109/C109M [45], a displacement-controlled loading method was employed at a rate of 0.2 mm/min. The compressive tests were conducted using the electro-hydraulic servo pressure testing machine, model C088-01, produced by MATEST (Bergamo, Italy). The load data were collected through the built-in sensors of the machine. Before loading, strain gauges were attached longitudinally to the cylindrical specimens to measure the longitudinal strains and determine the elastic modulus. Additionally, two linear variable differential transformers (LVDTs) were set up to measure the axial displacements of the specimens throughout the loading process, with a gauge length of 50 mm. During loading, data for the displacement, strain, and load were synchronously collected using a TDS540 data acquisition system at a frequency of 1 Hz. The number of test samples was three. The specific experimental setup is illustrated in Figure 5a.

The axial tensile test, following JSCE (2008) [46], was conducted using a controlled loading method at a rate of 0.5 mm/min. The tensile tests were conducted using the YNS-Y3000 electro-hydraulic servo universal testing machine produced in China. The load data were collected through the built-in sensors of the machine. The specimen was secured at both ends with universal joints connected to the loading end of the testing machine to prevent eccentric tension during the test. Linear variable differential transformers (LVDTs) were placed on both sides of the specimen to measure the longitudinal displacements during tension, with a gauge length of 80 mm. Additionally, during the tensile test, the displacement data were collected using a TDS540 data acquisition system, while the load data were obtained directly from the universal testing machine, both at a frequency of 1 Hz. The number of test samples was three. The specific experimental setup is depicted in Figure 5b.

To analyze the microstructure and composition of the samples, we used a scanning electron microscope (SEM). During the testing, we employed the backscattered electrons (BSE) mode to enhance the resolution of the sample’s elemental composition and phase distribution. We prepared two cubic samples, each with an edge length of approximately 7 mm, from the fracture section of the tensile specimens. One sample was used to observe the fiber distribution and failure modes, with an acceleration voltage of 5 kV applied to avoid damage to the fibers by the electron beam. The other sample’s observation surface was polished to a smooth finish using silicon carbide sandpaper up to a 7000-grit level. Elemental distribution of the fiber–matrix interfacial transition zone (ITZ) was obtained using the EDS line scan mode at an acceleration voltage of 15 kV. For EDS testing, C, Ca, and Si were selected to differentiate between the fibers, matrix, and ITZ.

## 3. Results and Discussion

### 3.1. Flowability

**Figure 6:**
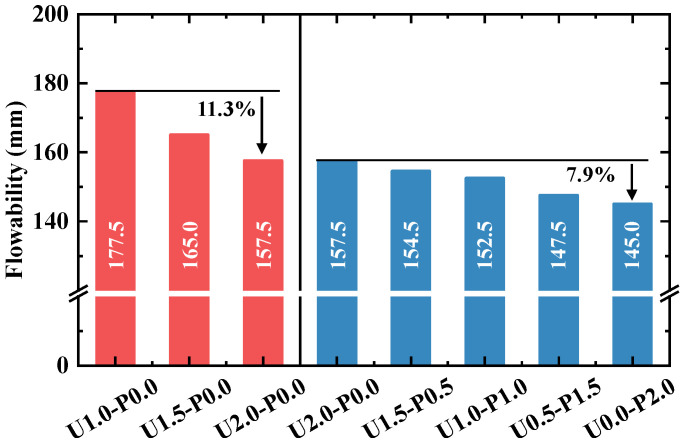
Flowability of fresh SHGC.

Figure 6 illustrates the flowability of fresh SHGC. As the volume fraction of the PE fibers increases from 1% to 2%, the flowability of the fresh SHGC paste decreases from 177.5 mm to 157.5 mm, marking an 11.3% reduction. This reduction is attributed to the tendency of flexible PE fibers to bend and interfere with the internal components of the fresh paste, thereby reducing the flowability [47]. Increasing the content of PE fibers exacerbates this negative effect. Furthermore, Figure 6 shows that increasing the replacement ratios of PVA fibers significantly reduces the flowability of the fresh SHGC paste. For instance, U0.0-P2.0 exhibits a 7.9% decrease in flowability compared to U2.0-P0.0. Unlike hydrophobic PE fibers, the hydroxyl groups on the surface of PVA fibers may form physical–chemical bonds with the polar geopolymer matrix [48], potentially increasing the interaction between fibers and the matrix, thereby reducing the paste’s flowability. Optimal flowability is crucial for minimizing internal defects in SHGC. Therefore, selecting appropriate fiber volume fractions and PVA replacement ratios is essential.

### 3.2. Compressive Behavior

#### 3.2.1. Failure Mode of Compressive Specimens

**Figure 7:**
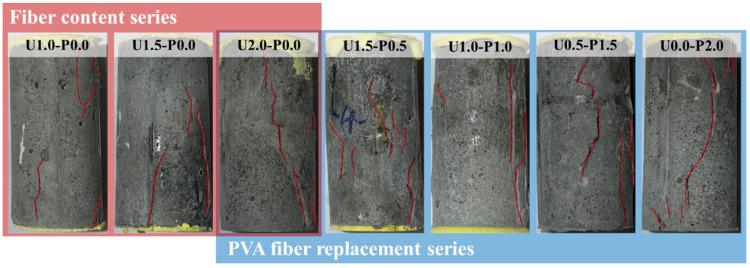
Compressive failure modes of SHGC.

Figure 7 depicts the compressive failure modes of the SHGC. We used red lines in the figure to depict and highlight the cracks. Under axial compressive loading, microcracks initiate and gradually propagate within the concrete, connecting and closing with an increasing load. As the load increases, these microcracks accumulate and form macroscopic cracks when the load reaches its peak. At this point, strain energy is suddenly released, causing the SHGC specimen to rapidly lose its load-bearing capacity. In Figure 7, it is evident that the primary cracks in the SHGC are predominantly oriented longitudinally, exhibiting a mode of failure characterized by longitudinal splitting, indicating significant brittleness. However, the U2.0-P0.0 specimen shows the smallest crack inclination angle, suggesting an oblique shear failure mode. This indicates that the inclusion of high-strength PE fibers at a volume fraction of 2% helps mitigate the compressive brittleness of the SHGC. Although the hydrophilicity of PVA fibers allows them to form a good interfacial bond with the matrix (as evidenced by the microscopic analysis in Section 3.3.1), their relatively low tensile strength makes them prone to rupture when macro-cracks develop in the compressive specimens. This, in turn, causes the SHGC to exhibit brittle failure characteristics.

#### 3.2.2. Compressive Stress–Strain Curves

**Figure 8:**
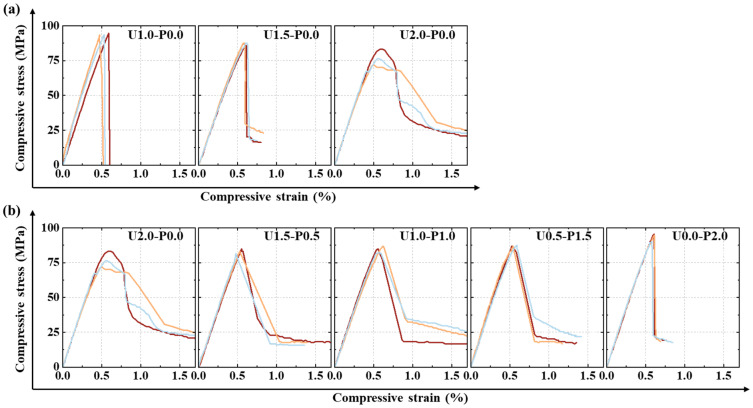
Compressive stress–strain curves of SHGC: (**a**) fiber content series; (**b**) PVA fiber replacement series.

Figure 8 depicts the compressive stress–strain curves of the SHGC. In Figure 8, it can be observed that the stress–strain curves, except for U2.0-P0.0, exhibit a sudden drop in stress after reaching the peak, indicating significant brittle failure characteristics consistent with the longitudinal splitting failure mode. Figure 8a shows that as the volume fraction of PE fibers increases, the descending branch of the curve gradually becomes more gradual, albeit with a decrease in peak strength. Compared to the brittle behavior of U1.0-P0.0, which loses its load-bearing capacity immediately after peaking, U2.0-P0.0 shows a slow decline in stress after reaching the peak, indicating improved ductility. A sufficient fiber content effectively suppresses the development of internal defects in the specimen, thereby enhancing compressive ductility. Figure 8b indicates that as the replacement ratio of the PVA fibers increases, the descending branch of the curve becomes steeper, exhibiting brittle behavior similar to that observed with a low PE fiber content, albeit with an increase in peak strength. The limited strength of PVA fibers and their strong interface effect with the matrix make them more prone to fracture at the crack surfaces rather than pull-out failure (discussed in Section 3.3.1), leading to post-peak brittleness in the SHGC.

#### 3.2.3. Compressive Properties

From the compressive stress–strain curves in Figure 8, various performance parameters of SHGC can be obtained, including the peak strength, peak strain, and elastic modulus. The elastic modulus is calculated according to ASTM C469/C469M [49] (Equation (1)). The specific results are shown in Table 4. The values in parentheses represent the standard deviation.

(1) $$E=\frac{\sigma_{2}-\sigma_{1}}{\varepsilon_{2}-0.000050}$$

where, $\sigma_{1}$ is the stress corresponding to a longitudinal strain of 0.000050; $\sigma_{2}=0.4\sigma_{CM}$, $\sigma_{CM}$ is the peak stress; $\varepsilon_{2}$ is the strain corresponding to $\sigma_{2}$.

**Figure 9:**
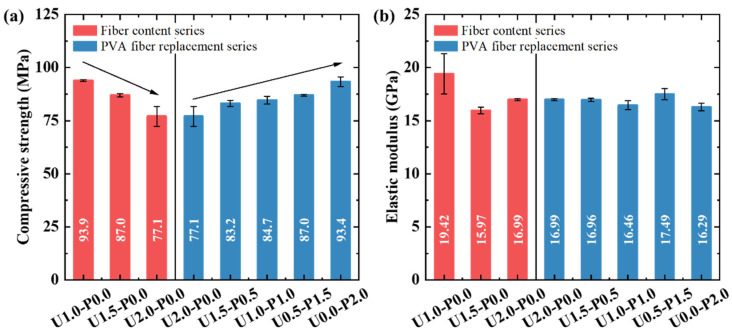
Compressive performance of SHGC under different PE fiber contents and PVA fiber replacement ratios. (**a**) Compressive strength; (**b**) elastic modulus.

Figure 9a shows the compressive strength of the SHGC under different PE fiber contents and PVA fiber replacement ratios. With an increasing PE fiber content, the compressive strength of the SHGC decreases approximately linearly. Compared to U1.0-P0.0, U2.0-P0.0 exhibits a decrease in compressive strength by 17.9%. This is attributed to a significant reduction in flowability when the PE fiber content reaches 2%, potentially increasing internal defects in the SHGC [50]. Additionally, the weaker synergistic interaction between the hydrophobic PE fibers and the matrix before cracking, along with excessive PE fibers, may contribute to defects themselves, thereby leading to a decrease in compressive strength. In contrast, replacing PE fibers with PVA fibers effectively enhances the compressive strength. As the PVA fiber replacement ratio increases, the compressive strength gradually improves. Compared to U2.0-P0.0, U0.0-P2.0 shows an increase in compressive strength by 21.1%. This enhancement may be due to the strong interfacial bonding between PVA fibers and the matrix, which enhances their synergistic effect with the matrix and effectively restrains crack propagation, thereby improving the compressive strength of the SHGC [51].

Figure 9b presents the elastic modulus of the SHGC under different PE fiber contents and PVA fiber replacement ratios. Increasing the PE fiber content adversely affects the elastic modulus, while the replacement of PE fibers with PVA fibers shows minimal impact on the elastic modulus. Sakulich and Li [52] and Lei et al. [53] found, through nanoindentation tests, that the lateral elastic modulus of PE and PVA fibers is much lower than that of the matrix. Therefore, the inherently lower elastic modulus of synthetic fibers is the primary reason for the reduction in the compressive elastic modulus of the SHGC.

### 3.3. Tensile Behavior

#### 3.3.1. Failure Mode of Tensile Specimens

**Figure 10:**
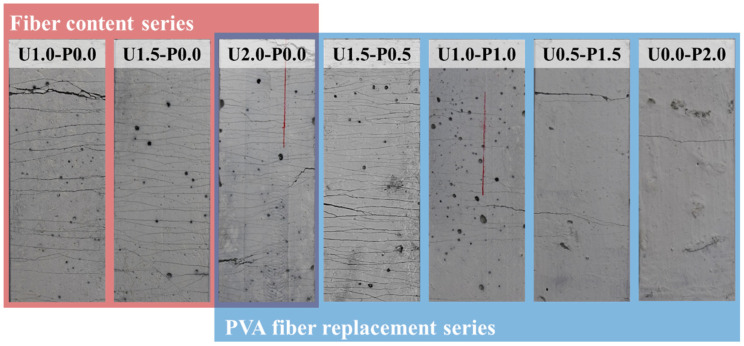
Typical Axial Tensile Failure Modes of SHGC Specimens.

Figure 10 illustrates the axial tensile failure modes of typical SHGC specimens. Under tensile loading, the SHGC initially cracks at the weakest section, followed by stress redistribution, where the tension is borne by the fibers. As the load increases, the SHGC continues to develop cracks, ultimately leading to a failure mode characterized by multiple crack propagation. The bridging capability of fibers directly influences the tensile failure mode [54]. With an increasing PE fiber content, the number of cracks gradually increases while the residual crack width decreases. Partial replacement of the PE fibers with PVA fibers results in a reduction in the number of cracks in the SHGC. When the PVA fiber replacement ratio exceeds 75%, specimens U0.5-P1.5 and U0.0-P2.0 exhibit a sharp decrease in crack number, indicating a pronounced brittle tensile failure mode.

**Figure 11:**
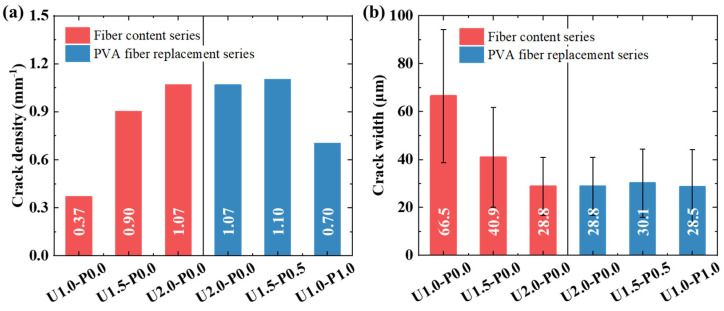
Crack characteristic parameters of typical SHGC specimens. (**a**) Crack density; (**b**) residual crack width.

Using a microscope (TD-2KH, produced by Sanqtid (Shenzhen, China), residual crack widths of the axial tensile failure specimens were measured, and the number of cracks and crack density (ratio of the crack number to measured length, mm^−1^) were statistically analyzed, as shown in Figure 11. It is noteworthy that specimens U0.5-P1.5 and U0.0-P2.0, which exhibited brittle failure, are not included in the statistics. Figure 11a presents the crack density of the SHGC. With an increasing PE fiber content, the crack density gradually increases. Compared to U1.0-P0.0, U2.0-P0.0 shows a 189% increase in crack density, indicating that an increasing fiber content effectively enhances the fiber bridging capability, which is consistent with the findings of Wang et al. [55]. However, replacing 50% of the PE fibers with PVA fibers results in a 65.4% reduction in crack density. Figure 11b displays the residual crack width of the SHGC. Increasing the PE fiber content effectively suppresses crack propagation, with U2.0-P0.0 having a residual crack width of only 43.3% compared to U1.0-P0.0. Replacement with PVA fibers has a minimal effect on the residual crack width.

**Figure 12:**
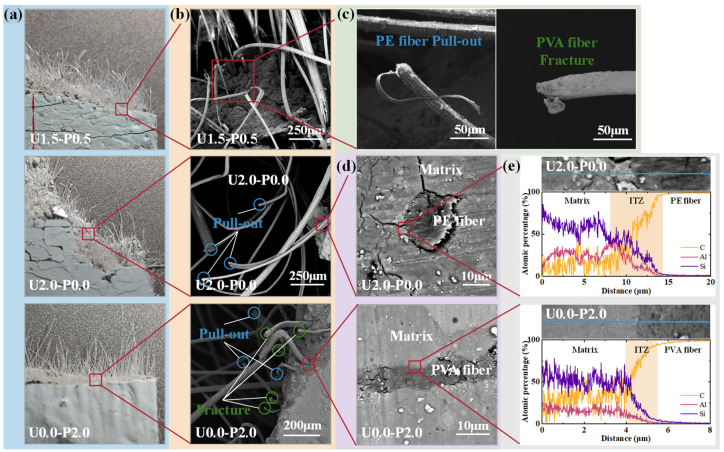
SHGC tensile specimen fracture surface microanalysis. (**a**) Fracture surface morphology of the specimen; (**b**) SEM images of the specimen fracture surface; (**c**) pull-out failure characteristics of PE fibers and fracture failure characteristics of PVA fibers; (**d**) microstructure of fibers, matrix, and ITZ (interfacial transition zone); (**e**) element distribution at fibers, matrix, and ITZ obtained by linear scanning using EDS.

Figure 12 shows the microstructural features of the SHGC tensile specimen fracture surfaces. Figure 12a displays the fracture surface morphology of specimens U1.5-P0.5, U2.0-P0.0, and U0.0-P2.0. It can be observed that the fiber distribution on the fracture surface of U0.0-P2.0 is sparser, indicating a tendency for PVA fibers to undergo fracture failure at the crack surface, whereas PE fibers tend to experience pull-out failure due to their higher strength. Figure 12b presents the micrographs of the fiber distribution at the fracture surface of specimens U1.5-P0.5, U2.0-P0.0, and U0.0-P2.0. It is evident that the fracture surface of U2.0-P0.0 predominantly exhibits pull-out failure of the PE fibers, whereas a significant proportion of the PVA fibers undergo fracture failure at the fracture surface of U0.0-P2.0. This contributes to the reduced saturation cracking performance of the SHGC with an increasing PVA fiber content. Figure 12c shows an enlarged view of the fiber ends at the fracture surface of specimen U1.5-P0.5, which was mixed with both PE and PVA fibers, revealing the pull-out and fracture failures of PE and PVA fibers, respectively. Figure 12d illustrates the microstructure of the fibers and matrix, showing a loose interface transition zone (ITZ) between the PE fibers and the matrix, whereas the PVA fibers, which possess hydrophilic characteristics, exhibit tight bonding with the matrix. Linear scans of the fiber–matrix ITZ using an energy-dispersive spectrometer (EDS), distinguishing elements C, Al, and Si, are shown in Figure 12e. In Figure 12e, it can be observed that the ITZ width between the PVA fibers and the matrix is smaller compared to that between the PE fibers and the matrix, indicating better interface properties between the PVA fibers and the matrix, enabling an improved stress transfer and synergistic effects with the matrix.

#### 3.3.2. Tensile Stress–Strain Curves

**Figure 13:**
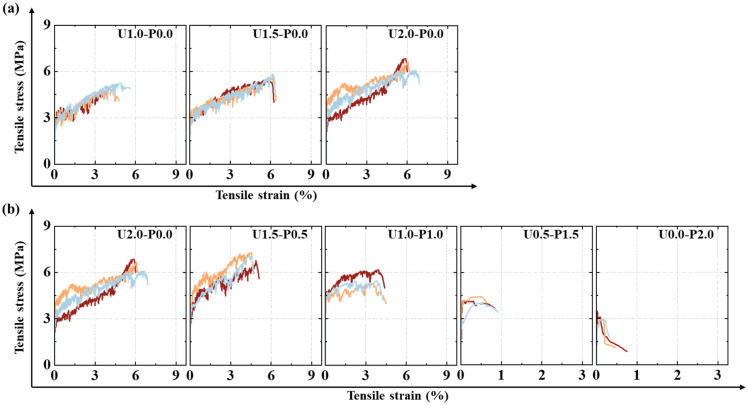
SHGC tensile stress–strain curves: (**a**) Fiber content series; (**b**) PVA fiber replacement series.

Figure 13 shows the stress–strain curves of the SHGC. In Figure 13, it can be seen that, except for U0.5-P1.5 and U0.0-P2.0, which experienced brittle failure, the stress–strain curves of the SHGC exhibit strain-hardening after cracking, accompanied by “sawtooth” fluctuations due to stress redistribution. Figure 13a indicates that with an increase in the PE fiber volume fraction, both tensile strength and ultimate tensile strain significantly improve. Figure 13b demonstrates that with a 25% substitution of PVA fibers, the tensile strength increases, but the tensile strain decreases noticeably. Further increasing the substitution rate of PVA fibers to 50% enhances the initial cracking strength but results in a significant decrease in tensile strength and ultimate tensile strain. The tensile strain energy density is represented by the area under the stress–strain curve and is calculated using Equation (2):

(2) $$g_{se}=\int_{0}^{\varepsilon_{u}}\sigma \left(\varepsilon \right)d\varepsilon$$

#### 3.3.3. Tensile Properties

From the tensile stress–strain curves in Figure 13, the tensile performance parameters of the SHGC can be obtained, including the initial cracking strength ($\sigma_{c}$), initial cracking strain ($\varepsilon_{c}$), tensile strength ($\sigma_{u}$), ultimate tensile strain ($\varepsilon_{u}$), and tensile strain energy density ($g_{se}$). The specific results are presented in Table 5.

**Figure 14:**
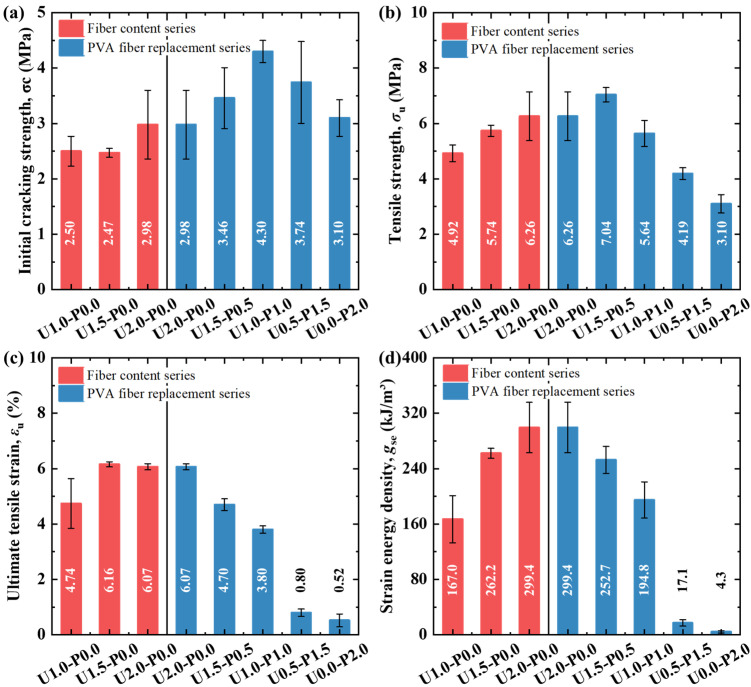
Tensile performance indicators of SHGC. (**a**) Initial cracking strength; (**b**) tensile strength; (**c**) ultimate tensile strain; (**d**) strain energy density.

Figure 14 presents the tensile performance parameters of the SHGC under different PE fiber contents and PVA fiber replacement rates. Figure 14a displays the initial cracking strength of the SHGC under various fiber parameters. As the PE fiber content increases, there is a rising trend in the initial cracking strength, with U2.0-P0.0 showing a 19.2% increase compared to U1.0-P0.0. Partial or complete replacement of the PE fibers with PVA fibers effectively enhances the initial cracking strength, exhibiting a trend of increase followed by a decrease. Due to the high tensile modulus of PE fibers reaching 120 GPa—much higher than the matrix—increasing their content can enhance the initial cracking strength. The hydrophilicity of PVA fibers allows for better synergistic effects with the matrix before cracking occurs in the SHGC, effectively increasing the initial cracking strength. The initial cracking strength of U1.0-P1.0 reaches 4.3 MPa. However, excessive replacement of the PVA fibers may introduce defects due to a loss of flowability, thereby negatively affecting the initial cracking strength, with a decrease observed after a replacement rate of 50%. Additionally, Figure 14a indicates that when the overall fiber volume content is 2% (including the different PVA fiber replacement rates), the initial cracking strength of the SHGC exhibits significant dispersion. The initial cracking strength in tension is primarily controlled by the matrix. The increased internal porosity and its uneven distribution at high fiber volume fractions lead to a greater dispersion in the matrix’s initial cracking strength.

Figure 14b shows the tensile strength of the SHGC under different fiber parameters. The variation trend of tensile strength is similar to that of the initial cracking strength, except that the tensile strength begins to decrease after a PVA fiber replacement ratio of 25%. The lower strength of PVA fibers and their propensity to fracture rather than pull out at the SHGC cracks (as noted in Section 3.3.1) contribute to the degradation of tensile strength. Unlike the initial cracking strength, the tensile strength at the ultimate state is primarily controlled by the fiber’s bridging performance. The high viscosity of the matrix ensures good fiber dispersion uniformity, allowing the tensile strength of the SHGC to exhibit good consistency.

Figure 14c and Figure 13d, respectively, illustrate the ultimate tensile strain and tensile strain energy density of the SHGC under different fiber parameters. The SHGC with 2% PE fibers exhibited the highest tensile deformation capability and strain energy density. PVA fibers tend to fracture prematurely during tension, reducing both the deformation and energy absorption capabilities, as evidenced by the consistent patterns in the residual crack number and width changes.

### 3.4. Economic and Environmental Analyses

Reducing the content of PE fibers and partially replacing them with cost-effective PVA fibers can enhance the economic and environmental sustainability of SHGCs. Furthermore, analysis of the experimental results indicates that both methods positively impact certain mechanical properties. Therefore, considering mechanical performance better reflects the influence of the fiber parameter variations on the economic and environmental performance of the SHGC.

**Figure 15:**
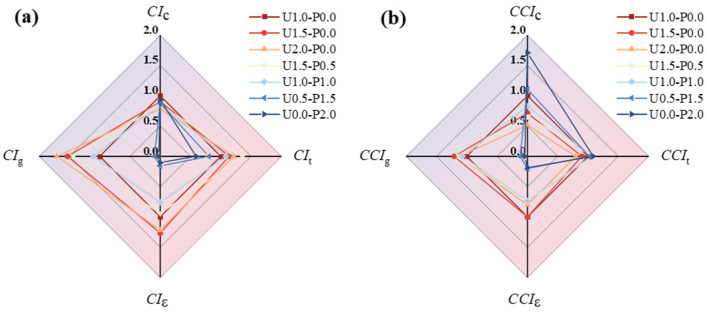
Radar chart analyses of comprehensive carbon emissions and cost indices of SHGC under different fiber parameters. (**a**) Comprehensive carbon emission indices; (**b**) comprehensive cost indices.

Table 6 presents the carbon emissions and material costs used for the calculations, with the carbon emission factor for barium chloride currently unverifiable and therefore not considered. The material costs were based on the local purchase prices and converted to USD using the exchange rate from July 2024. The carbon emission index (CI) and comprehensive cost index (CCI) [56] were employed as environmental performance assessment metrics, calculated using Equations (3) and (4), respectively. Table 7 and Figure 15 summarize the comprehensive carbon emissions and costs of the SHGC per cubic meter under different fiber parameters, where both the carbon emissions and cost indices have been normalized.

(3) $$CI_{i}=\frac{M_{i}}{CE}$$

(4) $$CCI_{i}=\frac{M_{i}}{\mathrm{Cos}t}$$

In this context, *M*_i_ represents the mechanical performance indicators, including compressive strength (MPa), tensile strength (MPa), ultimate tensile strain (%), and tensile strain energy density (kJ/m^3^).

From Figure 15a, it is evident that increasing the PE fiber content and partially replacing the PE fibers with PVA fibers results in higher comprehensive cost indices. Specifically, U1.5-P0.0 with 1.5% and U2.0-P0.0 with 2% PE fibers, as well as U1.5-P0.5 with 25% PVA fiber replacement, exhibit significantly higher cost-effectiveness in terms of the enveloping area on the radar chart. Each of these configurations shows different mechanical performance advantages, reflecting varying strengths in the comprehensive cost index. Figure 15b illustrates that U1.0-P0.0, using 1% PE fibers, excels in comprehensive environmental indices, with the largest enveloping area on the radar chart, indicating optimal comprehensive environmental benefits. However, due to PVA’s inherently higher carbon emission factor, the environmental benefits of partially or completely replacing the PE fibers with PVA fibers in the SHGC are not prominently enhanced. This effect becomes more pronounced when the PVA fiber replacement rate exceeds 75%, as indicated by a significant reduction in the radar chart’s enveloping area. Comprehensive economic and environmental indices are not the sole criteria for decision-making. When designing SHGC compositions, it is crucial to consider the actual requirements and balance the mechanical performance, economic benefits, environmental impacts, and other performance indicators in a multidimensional manner [54].

## 4. Conclusions

This paper investigates the axial compressive and tensile tests of strain-hardening geopolymer composites (SHGCs) with varying fiber parameters (the fiber volume fraction and PVA fiber replacement ratio). It explores the influence mechanisms of fiber parameters on static mechanical behaviors and comprehensively analyzes the economic and environmental benefits of the SHGC. The main conclusions obtained are as follows:

Increasing the volume fraction of high-performance PE fibers can effectively inhibit crack formation, thereby enhancing the post-peak ductility of the SHGC specimens under axial compression loads. However, increasing the volume fraction of PVA fibers results in brittle failure behavior under compression for the SHGC. This is because the relatively low strength of PVA fibers causes them to fracture at the cracked sections, and they are unable to effectively dissipate the strain energy during compressive failure.

The hydrophobic PE fibers have weak synergy with the matrix; an excessive amount of PE fibers can become defects themselves, leading to a degradation in compressive strength. The compressive strength of the SHGC decreased by 17.9% when the PE fiber content increased from 1% to 2%. In contrast, increasing the volume fraction of PVA fibers can effectively improve compressive strength due to their hydrophilic nature, which results in better interfacial bonding with the matrix, enhancing their synergistic action. When the PVA fiber replacement rate is 100%, the compressive strength is 21.1% higher than when the replacement rate is 0%.

Increasing the PE fiber content comprehensively improves the tensile properties of the SHGC, including the initial cracking strength, tensile strength, ultimate tensile strain, and tensile strain energy. However, increasing the PVA fiber replacement rate only improves the tensile strength of the SHGC (with a maximum tensile strength of 7.04 MPa at a 25% PVA fiber replacement rate) while negatively impacting the tensile deformation capacity and energy dissipation capacity. When the PVA fiber replacement rate exceeds 50%, the multiple cracking ability of the SHGC significantly declines, resulting in brittle tensile failure.

A comprehensive analysis of the production costs, carbon emissions, and mechanical performance indicators of the SHGC indicates that a PVA fiber replacement ratio of 25% achieves great comprehensive economic benefits with lower actual costs. The small difference in carbon emission factors between PVA and PE fibers means that the method of PVA fiber replacement has a minimal impact on carbon emission indices.

## Figures and Tables

**Table 1:** Chemical composition of GGBS and FA (unit: wt%).

Materials	Al_2_O_3_	SiO_2_	CaO	Fe_2_O_3_	K_2_O	SO_3_	MgO	TiO_2_	Other
FA	31.14	53.96	4.01	4.16	2.03	0	1.01	1.13	2.53
GGBS	17.70	34.50	34.00	1.03	0	1.64	6.01	0	5.12

**Table 2:** Physical properties of PE and PVA fibers.

Fiber Type	Density (g/cm^3^)	Strength (MPa)	Elastic Modulus (GPa)	Length (mm)	Diameter (μm)	Elongation at Break (%)
PE	0.97	2500	120	12	20	3.7
PVA	1.29	1830	40	12	15	6.9

**Table 3:** Mix proportions of SHGC (unit: kg/m^3^, with fibers represented by volume fraction).

Mix IDs	GGBS	FA	Quartz Sand	Alkali-Activator	Water	BaCl_2_	PE Fiber (%)	PVA Fiber (%)
U1.0-P0.0	849.7	364.1	242.8	242.8	72.8	12.1	1.0	0.0
U1.5-P0.0							1.5	0.0
U2.0-P0.0							2.0	0.0
U1.5-P0.5							1.5	0.5
U1.0-P1.0							1.0	1.0
U0.5-P1.5							0.5	1.5
U0.0-P2.0							0.0	2.0.

Note: The specimen grouping is represented in the Ux-Py format, where U stands for ultra-high molecular weight polyethylene (UHMWPE) fibers, P stands for PVA fibers, and x and y represent the volume fractions of the two fibers, respectively. For example, U1.0-P1.0 denotes a specimen containing 1.0% PE fiber and 1.0% PVA fiber.

**Table 4:** Compressive properties.

Mix IDs	Compressive Strength (MPa)	Peak Strain (%)	Elastic Modulus (GPa)
U1.0-P0.0	93.9 (0.49)	0.532 (0.05)	19.42 (1.90)
U1.5-P0.0	87.0 (0.83)	0.606 (0.01)	15.97 (0.32)
U2.0-P0.0	77.1 (4.69)	0.565 (0.05)	16.99 (0.10)
U1.5-P0.5	83.2 (1.43)	0.565 (0.02)	16.96 (0.16)
U1.0-P1.0	84.7 (1.80)	0.578 (0.03)	16.46 (0.42)
U0.5-P1.5	87.0 (0.41)	0.552 (0.03)	17.49 (0.52)
U0.0-P2.0	93.4 (2.30)	0.598 (0.01)	16.29 (0.35)

**Table 5:** Tensile properties.

Mix IDs	$\sigma_{c}$ (MPa)	$\varepsilon_{c}$ (%)	$\sigma_{u}$ (MPa)	$\varepsilon_{u}$ (%)	$g_{se}$ (kJ/m^3^)
U1.0-P0.0	2.50 (0.27)	0.024 (0.017)	4.92 (0.30)	4.74 (0.90)	167.0 (34.2)
U1.5-P0.0	2.47 (0.08)	0.027 (0.007)	5.74 (0.20)	6.16 (0.09)	262.2 (7.3)
U2.0-P0.0	2.98 (0.62)	0.069 (0.009)	6.26 (0.88)	6.07 (0.11)	299.4 (36.5)
U1.5-P0.5	3.46 (0.55)	0.057 (0.026)	7.04 (0.26)	4.70 (0.21)	252.7 (19.5)
U1.0-P1.0	4.30 (0.20)	0.050 (0.034)	5.64 (0.47)	3.80 (0.13)	194.8 (26.2)
U0.5-P1.5	3.74 (0.74)	0.032 (0.017)	4.19 (0.21)	0.80 (0.13)	17.1 (4.6)
U0.0-P2.0	3.10 (0.33)	0.025 (0.016)	3.10 (0.33)	0.52 (0.23)	4.3 (1.6)

**Table 6:** Carbon emission factors and material costs of raw materials.

Materials	GGBS	FA	NaOH	Na_2_SiO_3_	BaCl_2_	QS	PE Fibers	PVA Fibers	Water
Cost (USD/t)	48.7	26.4	2783.4	417.5	3340.1	55.7	55,667.6	6262.6	0.42
Carbon emission factor (kg CO_2_/kg)	0.019 [56]	0.009 [56]	1.915 [56]	0.804 [57]	-	0.010 [56]	2.000 [58]	1.710 [59]	0.8 [59]

**Table 7:** Comprehensive carbon emissions and cost indices (normalized) of SHGC under different fiber parameters.

Mix IDs	*Cost*	*CE*	*CI*_c_	*CI*_t_	*CI*_ε_	*CI*_g_	*CCI*_c_	*CCI*_t_	*CCI*_ε_	*CCI*_g_
U1.0-P0.0	1.00	1.00	1.00	1.00	1.00	1.00	1.00	1.00	1.00	1.00
U1.5-P0.0	1.30	1.02	0.90	1.14	1.27	1.53	0.90	1.00	1.21	0.90
U2.0-P0.0	1.60	1.05	0.78	1.21	1.22	1.71	0.80	0.80	1.12	0.80
U1.5-P0.5	1.34	1.05	0.84	1.36	0.94	1.44	1.07	0.74	1.13	1.07
U1.0-P1.0	1.09	1.06	0.85	1.08	0.76	1.10	1.05	0.74	1.07	1.05
U0.5-P1.5	0.84	1.06	0.87	0.80	0.16	0.10	1.02	0.20	0.12	1.02
U0.0-P2.0	0.58	1.06	0.94	0.59	0.10	0.02	1.08	0.19	0.04	1.08

Note: *CI*_c_, *CI*_t_, *CI*_ε_, and *CI*_g_, respectively, denote comprehensive carbon emission indices considering compressive strength, tensile strength, ultimate tensile strain, and tensile strain energy density. *CCI*_c_, *CCI*_t_, *CCI*_ε_, and *CCI*_g_, respectively, denote comprehensive cost indices considering compressive strength, tensile strength, ultimate tensile strain, and tensile strain energy density.

## Data Availability

The data presented in this study are available on request from the corresponding author. The data are not publicly available due to confidentiality issues.
